# Deep_KsuccSite: A novel deep learning method for the identification of lysine succinylation sites

**DOI:** 10.3389/fgene.2022.1007618

**Published:** 2022-09-29

**Authors:** Xin Liu, Lin-Lin Xu, Ya-Ping Lu, Ting Yang, Xin-Yu Gu, Liang Wang, Yong Liu

**Affiliations:** ^1^ School of Medical Informatics and Engineering, Xuzhou Medical University, Xuzhou, China; ^2^ College of Computer Science and Technology, China University of Mining and Technology, Xuzhou, China; ^3^ Laboratory Medicine, Guangdong Provincial People’s Hospital, Guangdong Academy of Medical Sciences, Guangzhou, China; ^4^ Jiangsu Center for the Collaboration and Innovation of Cancer Biotherapy, Cancer Institute, Xuzhou Medical University, Xuzhou, Jiangsu, China

**Keywords:** post-translational modification, lysine succinylation, deep learning, CNN, protein

## Abstract

Identification of lysine (symbol Lys or K) succinylation (Ksucc) sites centralizes the basis for disclosing the mechanism and function of lysine succinylation modifications. Traditional experimental methods for Ksucc site ientification are often costly and time-consuming. Therefore, it is necessary to construct an efficient computational method to prediction the presence of Ksucc sites in protein sequences. In this study, we proposed a novel and effective predictor for the identification of Ksucc sites based on deep learning algorithms that was termed as Deep_KsuccSite. The predictor adopted Composition, Transition, and Distribution (CTD) Composition (CTDC), Enhanced Grouped Amino Acid Composition (EGAAC), Amphiphilic Pseudo-Amino Acid Composition (APAAC), and Embedding Encoding methods to encode peptides, then constructed three base classifiers using one-dimensional (1D) convolutional neural network (CNN) and 2D-CNN, and finally utilized voting method to get the final results. K-fold cross-validation and independent testing showed that Deep_KsuccSite could serve as an effective tool to identify Ksucc sites in protein sequences. In addition, the ablation experiment results based on voting, feature combination, and model architecture showed that Deep_KsuccSite could make full use of the information of different features to construct an effective classifier. Taken together, we developed Deep_KsuccSite in this study, which was based on deep learning algorithm and could achieved better prediction accuracy than current methods for lysine succinylation sites. The code and dataset involved in this methodological study are permanently available at the URL https://github.com/flyinsky6/Deep_KsuccSite.

## 1 Introduction

Protein post-translational modification (PTM) is ubiquitous in various prokaryotes and eukaryotes, which also plays important roles in many biological processes involving diseases such as such as cancer, Alzheimer’s Disease (AD), and cardiovascular disease (Wu et al., 2019; Aggarwal et al., 2020; Ramesh et al., 2020). There currently have more than 450 known PTMs (Gao et al., 2020), among which phosphorylation, methylation, acetylation, succinylation, and ubiquitination have been extensively investigated. Lysine succinylation (Ksucc) is a type of newly discovered PTM, which was found to occur naturally on protein lysine residues *in vivo* (Zhang et al., 2011). Ksucc is the process in which a succinyl moiety covalently binds to lysine residues through enzymatic or nonenzymatic-dependent mechanisms. This modification adds negatively charged carboxyl groups to the modified site and neutralizes its positive charge, thus reconstructing the intra- and inter-molecule interactions, which may further affect the spatial structure of the protein and eventually lead to changes in the physicochemical properties of the modified protein (Zhang et al., 2011). Relevant studies have shown that Ksucc modification is widely involved in important physiological activities such as cell differentiation and cell metabolism (Alleyn et al., 2018), whose abnormalities are closely related to a variety of diseases, including cancer, metabolic diseases, neurological diseases. Therefore, identification of Ksucc sites is crucial to reveal its mechanisms, providing theoretical supports for the drug design and development of relevant diseases (Zhang et al., 2011; Park et al., 2013; Rardin et al., 2013).

Both experimental and computational methods have made significant contributions to the identification of Ksucc sites. In particular, experimental methods provide a large number of first-hand data for the study of Ksucc. However, the disadvantages are that these methods are time-consuming and expensive, which no longer meet the increasing needs of the fast-pacing research (Doll and Burlingame, 2015). Therefore, with the development of machine learning (ML) methods and the accumulation of Ksucc experimental data, more and more attentions have been put on computational methods with a focus on deep learning (DL) algorithms (Hasan et al., 2019). ML based method generally includes feature representation, feature selection, and algorithm application. SuccFind is the first Ksucc predictor that incorporated amino acid composition (AAC), the composition of k-spaced amino acid pairs (CKSAAP), and evolutionary-derived information to represent each peptide segment, after which F-score as feature reduction and SVM as a classifier were used to predict Ksucc sites (Xu et al., 2015). Then, pSuc-Lys (Jia et al., 2016), succiSite (Hasan et al., 2016), succiSite2.0 (Hasan et al., 2017), and GPSuc (Hasan and Kurata, 2018) were indepenenlty developed to predict Ksucc sites. Both pSuc-Lys and succiSite used random forest (RF) as the classifier but they differed in feature representation. That is, pSuc-Lys used general PseAAC to formulate peptide samples, while succiSite utilized the compositions k-spaced amino acid pairs (CKSAAP), binary, and amino acid index property as feature representation. In addition, succiSite2.0 took the composition of profile-based amino acid and orthogonal binary as features. GPsuc method adopted five features to encode sequence peptides. For each feature, the Wilcoxon rank was used as feature selection and RF was utilized as a base classifier, and finally logistic regression was used to integrate the results (Hasan and Kurata, 2018). There are also many other feature selection algorithms such as the minimum redundancy–maximum relevance (mRMR) and sequential forward selection (SFS) that were used for the prediction of lysine succinylation sites (Kao et al., 2020). In terms of feature representation, in addition to the physical and chemical properties, evolutionary information and structural information were also used in PSSM-Suc (Dehzangi et al., 2017), SSEvol-Suc (Dehzangi et al., 2018) and Success (López et al., 2018). SSKM_Succ was developed to solve the reliability of negative samples by using K-means (Ning, 2020). In 2022, Jia et al. (Jia et al., 2022) proposed the pSuc-FFSEA model, which not only used EBGW, one-hot, AAF_ DWT also adopted CBOW and CGR to encode amino acids, and then LASSO and two-layer stacked ensemble classifiers were utilized to construct the model. Although classical machine learning methods have contributed significantly to the prediction of Ksucc sites with good interpretability, it is difficult to obtain higher-level features by simple feature engineering, which limits the performance of the models to some extent.

Many deep learning-based Ksucc predictors have been proposed to further improve model performance by using their unique feature learning capabilities. Ning et al. (Ning et al., 2020) constructed a new tool named HybridSucc, which combined 10 types of informative features and implemented a hybrid-learning architecture by integrating deep-learning and conventional machine-learning algorithms into a single framework. Thapa et al. (2020) developed DeepSuccinylSite based on a convolutional neural network (CNN). Huang et al. (2021) proposed the LSTMCNNsucc model by combining long short-term memory (LSTM) and CNN. MDCAN_lys (Wang et al., 2021), which is a multilane dense convolutional attention network used the cascading model of dense convolutional block and convolutional block attention module to capture feature information at different levels. Zhang et al. (Zhang and Wang, 2022) constructed a mixed prediction model using ensemble learning strateg which established four basic classifiers LSTM-CNN, CNN-LSTM, LSTM, and CNN for five features of CKSAAP, ACF, BLOSUM62, AAindex, and one-hot, and then selected the classifier with the best performance for each feature, and finally integrated them. The biggest contribution of deep learning in Ksucc site prediction is that it can automatically extract high-dimensional features based on existing feature representations, and even directly extract features from amino acid sequences. Meanwhile, although the existing models have contributed much to the prediction of Ksucc sites, there is still more room for the method to be improved. In this study, we proposed a new Ksucc site predictor termed as Deep_KsuccSite, which is based on 1D-CNN, 2D-CNN, and voting methods. In this study, four representations including Enhanced Grouped Amino Acid Composition (EGAAC), the Composition of CTD (CTDC), Amphiphilic Pseudo-Amino Acid Composition (APAAC), and Embedding encoding were used to encode protein peptides. 1D-CNN and 2D-CNN were then used to construct base classifiers for 1D features and 2D features, respectively. Finally, output was obtained by voting on the results of each base classifier. In sum, we developed Deep_KsuccSite in this study, which was based on deep learning algorithm and could achieved better prediction accuracy than current methods for lysine succinylation sites. The code and dataset involved in this methodological study are permanently available at the URL https://github.com/flyinsky6/Deep_KsuccSite.

## 2 Materials and methods

**FIGURE 1:**
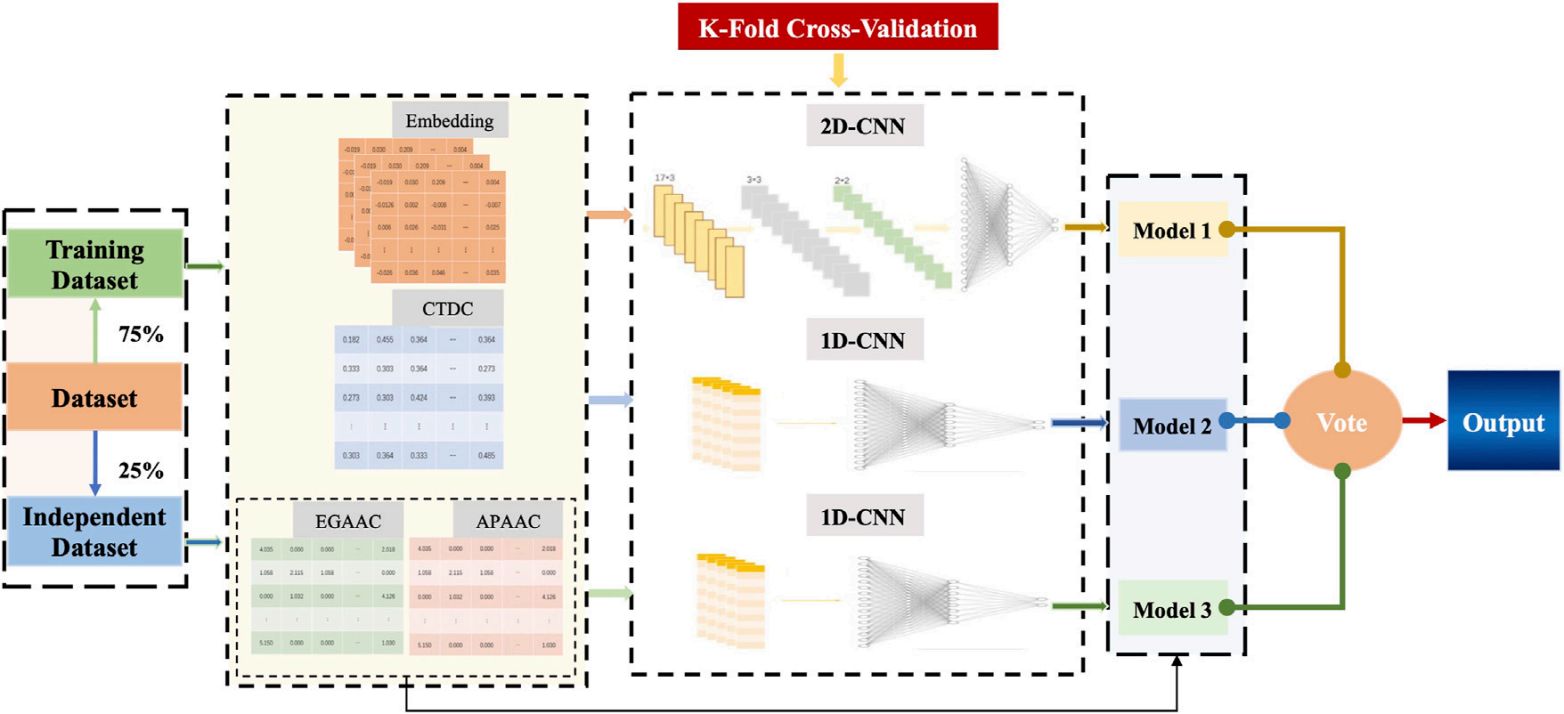
The overall framework of Deep_KsuccSite. The blue dashed line with arrows indicates the flow of the independent test dataset.

The direct fusion of feature information may cause mutual interference and weaken the quality of features, which in turn affects the effect of feature extraction. Therefore, in this paper, CNN was used as the base classifier of Deep_KsuccSite, that is, 2D-CNN for embedding features, and 1D-CNN for one-dimensional features such as CTDC and the combination of EGAAC and APAAC. Finally, the outputs of these three base models were voted to obtain the model output. The schematic illustration of the structure of Deep_KsuccSite method was shown in Figure 1. The major procedures for the development of Deep_KsuccSite could be summarized as following: 1) Data collection and preprocessing that were illustrated in Section 2.1; 2) Information encoding which were described in detail in Section 2.2; 3) classifiers module based on deep learning described in Section 2.3; 4) Performance evaluation and validation in Section 2.4.

### 2.1 Data collection and preprocessing

The Ksucc site data were downloaded from Protein Lysine Modification Database (PLMD,http://plmd.biocuckoo.org/) that was dedicated to protein lysine modifications (Xu et al., 2017). The PLMD database contains 18,593 Ksucc sites sourced from 6,378 protein sequences across 14 different species. In this study, the *Mus musculus* data were used to construct our model because it had the most Ksucc sites. Then, redundant protein sequences with high similarities for each species were strictly removed using CD-HIT with a threshold value of 0.4 to ensure squence quality and reduce sequence biases (Huang et al., 2010). Finally, a total of 932 protein sequences including 3,342 experimentally validated Ksucc sites were obtained as positive samples, and an equal amount of data from protein sequences without Ksucc site modification was obtained as negative samples by down-sampling technique (Supplementary Table S1). The length of each sample is L = 2N + 1, which was centered on lysine taking N amino acids to the left and right sides. For some peptides with lengths shorter than L, we filled them with pseudo-amino acids (denoted by the symbol *X*). The determination of the length L was described in Section 3.1. We randomly select 75% of the data set as the training set and the rest adata as an independent test set, which were used to train the model and evaluate the generalization ability of the model, respectively. Finally, 5,013 training datasets and 1,671 independent test datasets were obtained.

### 2.2 Information encoding

In order to construct a predictive model, peptides need to be transformed into feature vectors that can be recognized by machine learning algorithms. There are many methods for the vectorization of peptides used in the field of PTM, including physicochemical properties, evolutionary information, structural information and so on. In specificity, four encoding methods are considered in this paper, namely EGAAC, CTDC, APAAC, and Embedding Encoding. The first three methods were obtained by iLearn_plus (Chen et al., 2020), and all of four methods were briefly described as follows.

EGAAC calculates the enhanced grouped amino acid composition in a fixed-length window, sliding continuously from the N- to C-terminal of each peptide. Specifically, the 20 amino acids were classified into five categories based on different physicochemical properties (Lee et al., 2011): aliphatic group (g1:GAVLMI), aromatic group (g2:FYW), positive charge group (g3:KRH), negative charged group (g4:DE), uncharged group (g5:STCPNQ). The calculation formula is as follows:

$$G\left(g,n\right)=\frac{H\left(g,n\right)}{H\left(n\right)},g\in \left\{g1,g2,g3,g4,g5\right\},n\in \left\{w1,w2,\ldots ,wL\right\}$$ (1)

Where H(g,n) is the number of amino acids in group g within the window n, H(n) is the length of the window n. The fixed-length sequence window size defaults to 5 (Chen et al., 2018).

**CTDC** features represent the distribution patterns of amino acids for specific structural or physicochemical properties in a protein or peptide sequence. CTDC refers to the composition of CTD descriptors that are computed by the following procedures: 1) transforming amino acid sequences into sequences for structural or physicochemical properties; 2) according to Tomii and Kanehisa’s major amino acid index clustering, 20 amino acids were divided into three groups for each of the seven different physicochemical properties, detailed calculation of which could be seen in previous studies (Chen et al., 2020; Gu et al., 2020). In fact, CTDC has been successfully applied to the prediction of G protein-coupled receptors (Gu et al., 2020).

**APAAC** descriptor has the same form as the amino acid composition but contains more information related to the sequence order of the protein and the distribution of hydrophobic and hydrophilic amino acids along its chain.

**Embedding Encoding** method. The essence of the embedding encoding is word embedding, which is very important in the field of natural language processing (Grohe, 2020). It can help us find the relationship between words that are difficult to detect, and this idea is currently getting more and more attention in the protein field, because There are many analogies between amino acid sequences and natural languages. For example, sequences are regarded as sentences, and amino acids are regarded as words. Therefore, each amino acid can be vectorized by embedding representation, and finally the representation of the entire sequence can be obtained by integration. In particular, the 20 amino acid residues and one pseudo residue are first converted into integers from 0 to 20, and then a vector representation of each integer (length 21) is obtained by training through the embedding layer in Keras. Finally, each peptide is represented as a 33*21 two-dimensional matrix.

### 2.3 Base classifier

CNN, one of the representative algorithms of deep learning, is a feed-forward neural network with deep structure and convolution computation. Its powerful representation learning capability has led to successful applications in image processing, natural language processing, biological information, and other fields (Alom et al., 2019; Hesamian et al., 2019). According to the format of input data, CNN can be classified into 1D-CNN and 2D-CNN (Kiranyaz et al., 2021). In this study, two base classifiers based on 1D-CNN and 2D-CNN were constructed for different features.

#### 2.3.1 1D-CNN classifier

**FIGURE 2:**
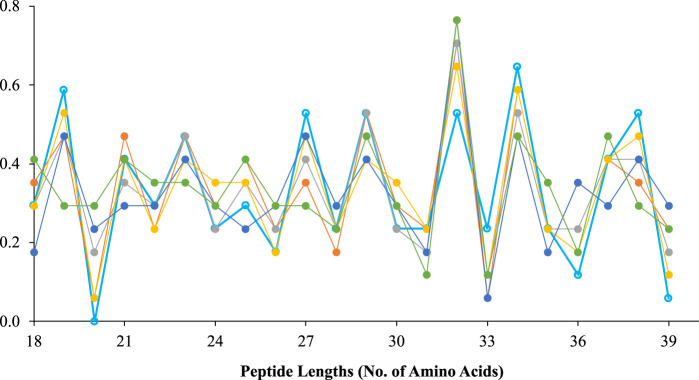
Schematic illustration of the sequence diagram of CTDC that showed the time sequence feature of the data type.

Traditional 2D-CNN are specialized for processing 2D data, such as images and videos. As an alternative, 1D-CNN has been recently developed (Kiranyaz et al., 2021). It has been shown that 1D-CNNs outperform 2D-CNNs in processing 1D signals in certain applications, e.g., patient EEG (Yildirim et al., 2018), high-power circuit, power engine fault detection (Eren et al., 2018), etc. In this study, CTDC, EGAAC and APAAC were 1D features with dimensions of 39, 24 and 145, respectively. Taking CTDC features as an example (Figure 2), it could be see that the positions of the 23rd, 27th, 29th, 32nd, and 34th features show obvious maximum values, and the positions of 20, 26, 28, 33, and 39 all show obvious minimum values. This suggests that they have they have good timing sequential characteristic and could thus be classified with 1D-CNN.

The structure of the 1D-CNN used in this paper mainly consisted of a Convolution Layer, a Dropout Layer, and a Fully-Connected Layer. Among them, there were 64 Convolution Layers with a step size of 2. In order to avoid overfitting, the Dropout Layer retained 40% of the connections, whilethe Fully-Connected Layer contained 32 units. Finally, the final output was calculated using the *SoftMax* activation function. Both convolutional and fully connected layers used rectified linear units (*ReLu*) as the activation function, and the optimizer was Stochastic Gradient Descent (SGD). The detailed structure and parameter settings were shown in Table 1, while the parameter range and settings of 1D-CNN were shown in Supplementary Table S2.

**TABLE 1:** Architecture and hyperparameter settings for 1D-CNN. The size column describes the kernel size of the convolutional layer, the size of the largest pooling layer, and the fully connected layer.

Layer no.	Layer type	Size	Activation
0	Input	L	—
1	CONV	64*2	Relu
6	Flatten	—	—
	Dropout	0.4	
7	Fc1	32	Relu
8	Output	2	SoftMax
9	Optimizer	SGD	

#### 2.3.2 2D-CNN classifier

**FIGURE 3:**
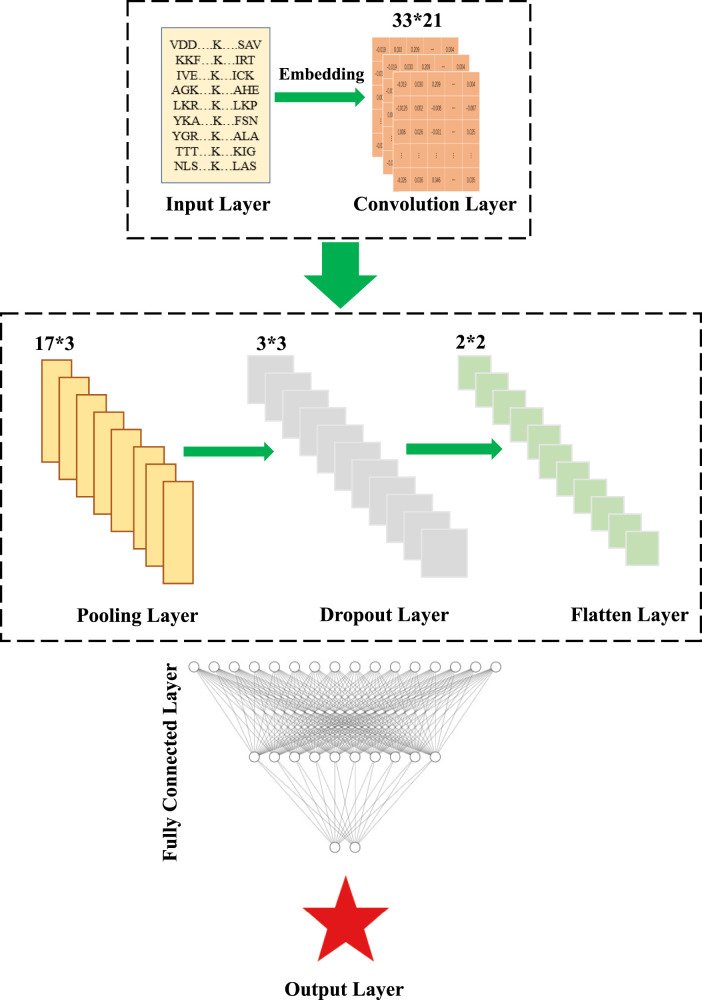
Schematic illustration of the architecture of the 2D-CNN, which contains an Input Layer, a Convolution Layer, a Pooling Layer, a Dropout Layer, a Flatten Layer, a Fully-Connected Layer, and an Output Llayer.

In this section, considering the advantages of CNN for feature extraction of 2D data such as images, 2D-CNN is used to construct a classifier for embedding encoding to reduce information loss during feature propagation, which was first proposed in previous study (Thapa et al., 2020). The framework of 2D-CNN was shown in Figure 3, which included an Input Layer, a Convolution Layer, a Pooling Layer, a Dropout Layer, a Flatten Layer, a Fully-Connected Layer, and an Output Layer. In this paper, we used 17*3 and 3*3 matrices with sliding windows for convolutional operations and used *ReLu* as the activation function for the normalized results.

To improve the operation efficiency and reduce the risk of overfitting, the Maxpooling Layer and Dropout Layer were embedded in the convolution module. Since the probability distribution of all classes needs to output, the Flatten Layer achieves the transition from the Convolutional Layer to fully-connected Layer by converting the matrices generated by Convolutional Layers into a vector. If the operations of the convolution, pooling, and activation function layers are understood as mapping the original data to the feature space of the hidden layer, then the fully-connected layer plays the role of mapping the learned “distributed feature representation” to the sample marker space. Here, two fully-connected layers in our DL model, denoted as Fc1 and Fc2, had 768 and 256 neurons, respectively. *ReLu* was also used as the activation function. Finally, the *SoftMax* activation function was used in the output layer to calculate the final output. The hyperparameter settings used for each layer were shown in Table 2.

**TABLE 2:** The hyperparameter settings of each 2D-CNN layer.

Layer no.	Layer type	Size	Activation
0	INPUT	33*21	—
1	CONV	64*17*3	Relu
2	MaxPooling	2*2	-
3	CONV	128*3*3	Relu
4	MaxPooling	2*2	—
5	Dropout	0.5	—
6	Flatten	—	—
7	Fc1	768	Relu
	Dropout	0.5	
8	Fc2	256	Relu
	Dropout	0.5	
	Output	2	Softmax
9	Optimizer	Adam	

### 2.4 Performance evaluation

To evaluate the performance of Deep_KsuccSite, we adopted several common statistical methods in this paper, including accuracy (Acc), sensitivity (Sen), precision (Pre), Matthew’s correlation coefficient (MCC) and F1 score. Detailed definitions were given below:

$$Acc=\frac{TP+TN}{TP+FP+TN+FN}$$ (2)

$$Sen=\frac{TP}{TP+FN}$$ (3)

$$Pre=\frac{TP}{TP+FP}$$ (4)

$$F1=\frac{2\times Pre\times Sen}{Pre+Sen}$$ (5)

$$MCC=\frac{\left(TP\times TN\right)-\left(FP\times FN\right)}{\sqrt{\left(TP+FN\right)\times \left(TN+FP\right)\times \left(TP+FP\right)\times \left(TN+FN\right)}}$$ (6)

Here, TP means the number of correctly predicted positive samples. TN means the number of correctly predicted negative samples. FP means the number of incorrectly predicted positive samples. FN means the number of incorrectly predicted negative samples (Crooks et al., 2004).

When the data set is balanced, accuracy indicates the percentage of the correctly predicted outcomes in the total sample. Sen refers to the percentage of true positive samples correctly classified, Pre refers to the probability of actually being positive among all predicted positive samples, F1 is the harmonic mean of Pre and Sen, MCC is essentially a correlation coefficient describing the correlation between the actual category and the predicted category, and it takes values in the range [-1,1] (Forbes, 1995). In addition, the receiver operating characteristic curve (ROC) and the area under ROC curves (AUC) were also used to assess the performance. OC calculates the range of sensitivities and specificities by setting different thresholds for continuous variables, which is a composite indicator of sensitivity and specificity (Fawcett, 2006). The average AUC value shows the overall performance, with larger values being better (Lobo et al., 2008).

## 3 Results and discussion

### 3.1 Selection of window size

**FIGURE 4:**
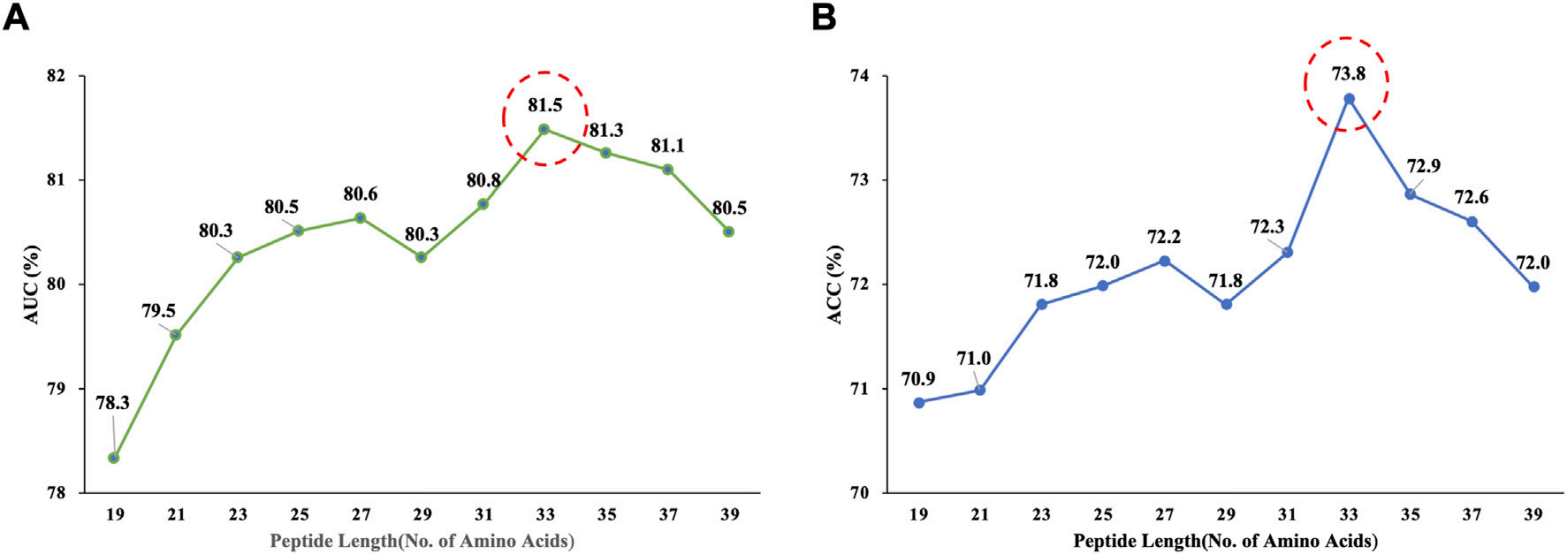
The Acc and AUC values corresponding to different window sizes of Deep_KsuccSite on the training data set. **(A)** AUC corresponding to window size. **(B)** ACC corresponding to window size. For both of the two parameters, highest values were achieved when the window size reached to 33.

The choice of window size has a direct impact on the performance of Deep_KsuccSite. If the window is too small, it is easy to ignore the global nature. Considering that the window with lengths greater than 40 may form structural domains and lead to model bias (Taylor, 1999), existing studies use windows in the range of 21–51 (Ning et al., 2020; Zhang et al., 2020; Zhu et al., 2020; Huang et al., 2021; Tasmia et al., 2021; Wang et al., 2021). Therefore, we analyzed the model performance when the length was between 21 and 39. The Acc and AUC values corresponding to the different windows of the Deep_KsuccSite on the training data set were shown in Figure 4. It could be seen that the highest values were obtained when the window reached 33 for both AUC (81.5%) and Acc (73.8%), respectively.

### 3.2 Performance evaluation and comparison

**FIGURE 5:**
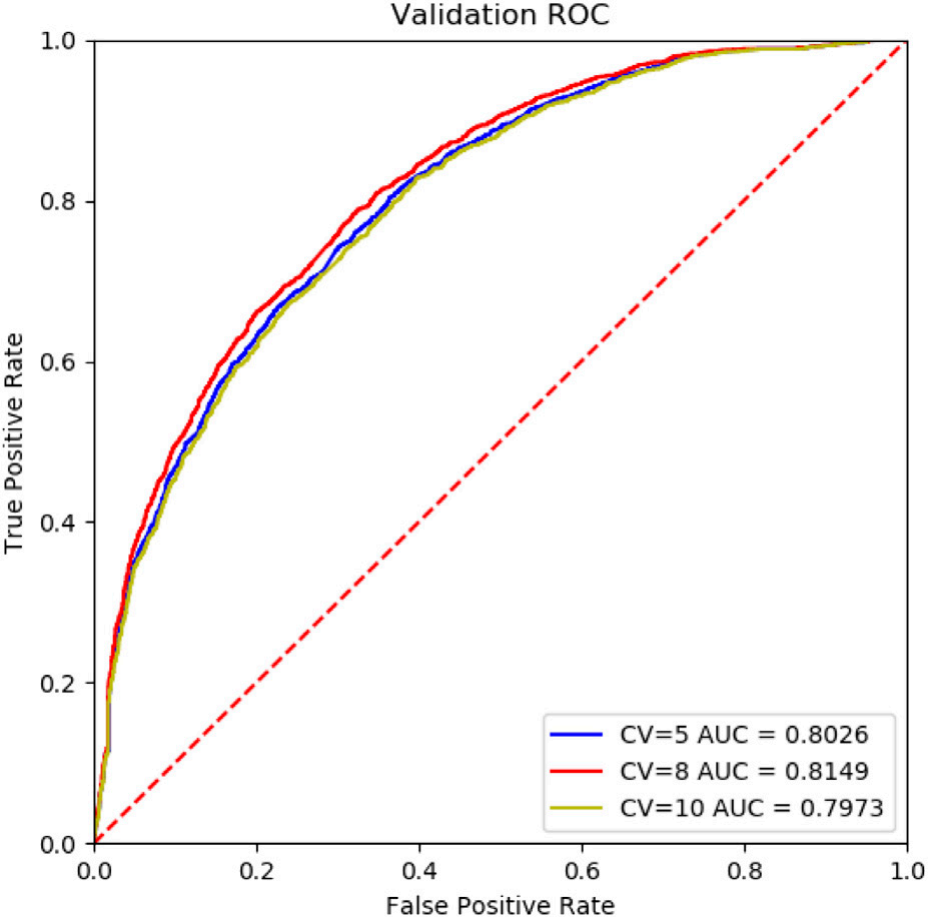
The comparision ROC curves of 5-,8-,10-fold cross-validation of the Deep_KsuccSite on the training data set. Blue, red and yellow curves indicated the ROC curves of 5-,8-,10-fold cross-validation, which had AUCs of 0.8026, 0.8149, and 0.7973, respectively.

To evaluate the performance of Deep_KsuccSite, 5-, 8- and 10-fold cross-validations were performed on the training dataset. The ROC curves for n-fold cross-validations were shown in Figure 5. The results showed that the AUC values were 0.8026, 0.8149, and 0.7973 for 5-,8-, and 10-fold cross-validations, respectively. The high consistency of different cross-validation results indicated the robustness of Deep_KsuccSite.

To verify the generalization capability of Deep_KsuccSite, the performance of Deep_KsuccSite was compared with other reported and publicly available Ksucc predictors. Although many servers or source code were released along with previous studies, only a few were available. In this study, four models were used to compare with Deep_KsuccSite, namely pSuc-FFSEA (Jia et al., 2022), DeepSuccinyISite (Thapa et al., 2020), SuccinSite (Hasan et al., 2016), and GPSuc (Hasan and Kurata, 2018). Among them, Both GPSuc and SuccinSite used Random Forest, and GPsuc developed generic and 9 species-specific Ksucc site classifiers by aggregating multiple complementary features, while SuccinSite was developed by integrating three sequence encoding methods. DeepSuccinyIsite proposed a novel embedding encoding to represent peptide segments based on CNN. Since most of the methods only provided web servers, we evaluated them on the independent test set, and the comparison results were presented in Table 3, in which the Pre of Deep_KsuccSite was only slightly lower than the Pre of DeepSuccinyIsite by 0.36%. Except for that, Deep_KsuccSite outperformed all the other methods in terms of the evaluation indices including Acc, Sen, Pre, F1, MCC, and AUC values.

**TABLE 3:** Comparison of Deep_KsuccSite with existing predictors of GPSuc, SuccinSite, and DeepSuccinyIsite on the independent test data.

Method	Acc(%)	Sen(%)	Pre(%)	F1 (%)	MCC	AUC(%)
GPSuc (Hasan and Kurata, 2018)	51.58	35.05	52.84	42.14	4.54	—
SuccinSite (Hasan et al., 2016)	56.38	29.31	64.42	40.29	16.05	—
DeepSuccinyIsite (Thapa et al., 2020)	69.42	67.84	**70.76**	69.27	38.90	69.44
pSuc-FFSEA (Jia et al., 2022)	58.93	37.93	68.75	48.89	21.47	59.71
Deep_KsuccSite	**71.87**	**77.03**	70.40	**73.57**	**43.85**	**78.03**

Note: Bold number means the best value achieved for a specific parameter when compared all the methods in the table.

### 3.3 Ablation experiments

Deep_KsuccSite is a model obtained by voting on different features or feature combinations corresponding to base classifiers, so the voting strategy, feature combination method, and base classifier are all factors that affect the performance of the model, and we conduct 3 types of ablation experiments on independent test data respectively.

#### 3.3.1 Voting ablation experiment

**FIGURE 6:**
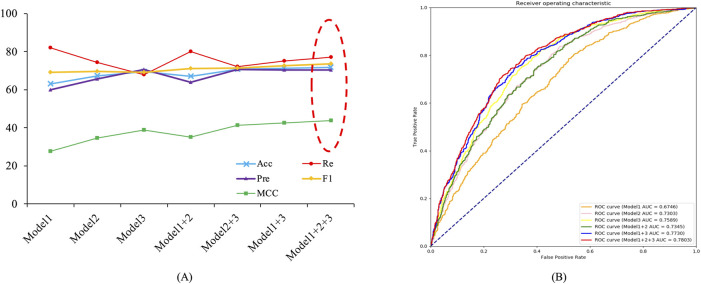
Line charts of voting results for different base models on independent test data. Model 1 denoted base classifier based on CTDC, Model 2 denoted base classifier based on the combination of APAAC and EGAAC, and Model 3 denotee based classifier based on embedding encoding. **(A)** Voting results for different combinations of models. **(B)** ROC curves and AUC values for different combinations of models.

The Deep_KsuccSite model was obtained by voting on the three base classifiers. To demonstrate the effectiveness of voting, we compared the model performance using different voting strategies for the base classifiers separately on independent test data, and the results were shown in Figure 6 and Supplementary Table S3. As one can see in Figure 6, the performance of the models obtained from different voting strategies varied slightly on the independent test data. Among them, the model voting on the three models achieved the best performance in almost all evaluation metrics with 71.87%, 70.40%, 73.57%, 43.85%, and 78.03% for Acc, Pre, F1, MCC, and AUC, respectively. It was noteworthy that CTDC-based Model 1 had the best Re with a value of 82.10%.

#### 3.3.2 Feature combination ablation experiment

**FIGURE 7:**
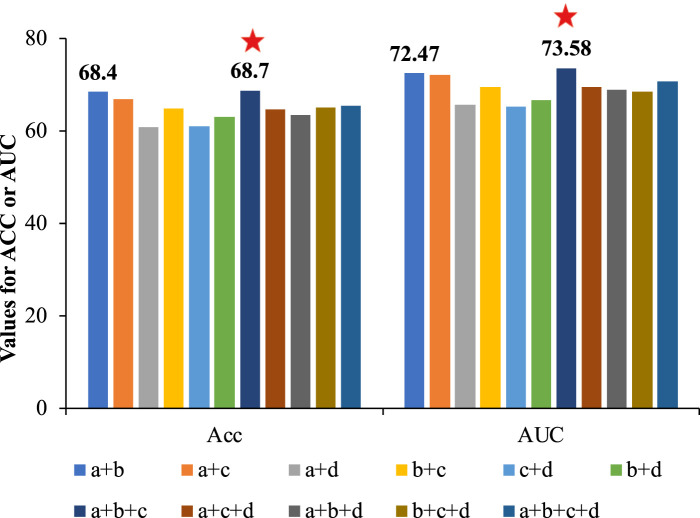
The comparison of Acc and AUC of different feature combinations based on CNN. Feature a denoted APAAC, feature b denotes CTDC, feature c denoted EGAAC, and feature d denoted embedding encoding. According to the results, the combination of a, b and c (EGAAC, APAAC and CTDC) showed the best performance.

Many studies improved the model performance by combining multiple features, but we speculated that direct information fusion might cause mutual interference, weakening feature quality and affecting model performance. To verify this speculation, we compared the performance of different feature combinations on independent test data, and the results were shown in Figure 7 and Supplementary Table S4.

**FIGURE 8:**
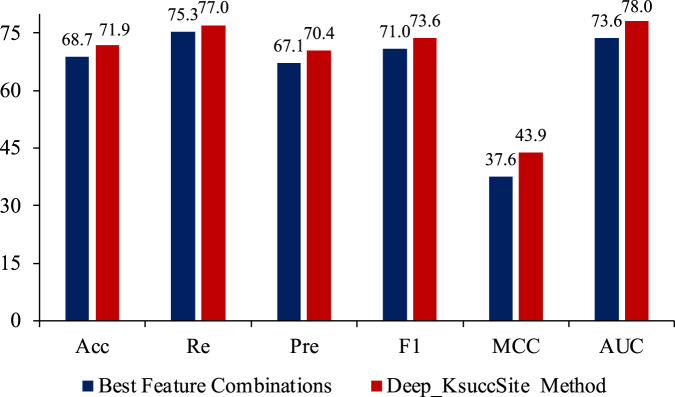
The comparison of best feature combinations (EGAAC, APAAC and CTDC) and Deep_KsuccSite method on independent test data. According to the results, the Deep_KsuccSite method showed consistent better performance than the best feature combinations.

As seen in Figure 7, there was no significant correlation between the number of features and the performance. Among these feature combinations, the combination of EGAAC, APAAC and CTDC (dark blue bars in Figure 7) had the best performance, while the performance of the combination of all four features was not outstanding and lower than many other feature combinations. Deep_KsuccSite effectively avoided this problem by selecting the best model for each class of special and then integrating the results of each model. For this reason, we compared the above optimal feature combinations with Deep_KsuccSite on independent test data, the results of which were shown in Figure 8.

As shown in Figure 8, Deep_KsuccSite outperformed the best combination found in Figure 7 on all evaluation metrics, especially on the MCC index. This further confirmed that simply integrating multiple features did not fully utilize the information of each feature. Choosing the appropriate model for each feature to integrate could improve the overall performance.

#### 3.3.3 Model architecture ablation experiment

As mentioned above, Deep_KsuccSite used CNN as the base classifier. To verify the effectiveness of CNN, we replaced it with SVM and LSTM. Among them, SVM is a classical machine learning model, which is good at dealing with small sample high-dimensional data and successfully applied in many PTM prediction studies (Ju et al., 2016; Chou, 2019), and LSTM is a RNN model that is good at dealing with time-series data. For the four features studied in this paper, the embedding feature needed to be vectorized into 1D features before use. The SVM classifier used kernel function, the parameters c and g were determined by five-fold cross-validation and grid search. LSTM used *SoftMax* as the activation function, and the remaining parameters were obtained by training. For a fair comparison, we used the same training and independent test data for these three models. Their comparison on the independent test data was shown in Table 4.

**TABLE 4:** Performance of different model architectures on the independent test data.

Model	Acc	Re	Pre	F1	MCC	AUC
SVM	67.32	68.67	67.56	68.11	34.62	74.26
LSTM	64.71	63.10	64.95	64.01	29.22	70.81
CNN	**71.87**	**77.03**	**70.40**	**73.57**	**43.85**	**78.03**

Note: Bold number means the best value achieved for a specific parameter when compared all the methods in the table.

As shown in Table 4, Deep_Ksucc outperformed the model based on SVM and LSTM in all evaluation metrics, with SVM coming second, and LSTM probably being the least suitable for those features. The main reason may be that large amount of information was lost when the embedding features were directly transformed into 1D data, and also many features did not have obvious temporal characteristics, so neither SVM nor LSTM could obtain better results.

### 3.4 Biological insights into ksucc prediction

**FIGURE 9:**
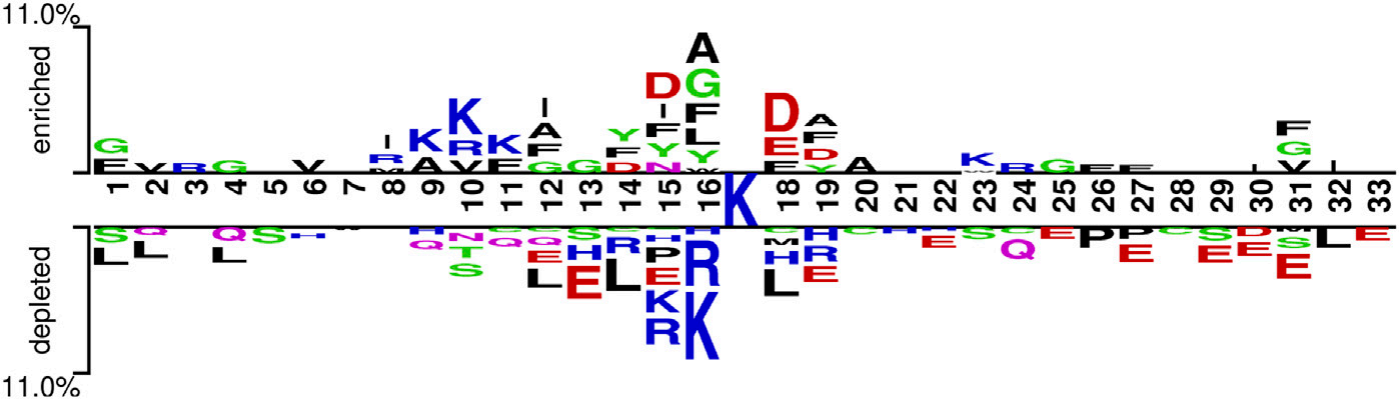
The statistical two-sample logo analysis with *t*-test on the samples (*p*-value < 0.05).

To further observe the differences between Ksucc and non-Ksucc peptides, two Sample logos with *t*-test (*p*-value < 0.05) was used to analyze the frequency of sequence occurrence at each position (Crooks et al., 2004). As seen in Figure 9, there was a significant difference in sequence preferences between Ksucc and non-Ksucc peptides. Aspartic acid (D), phenylalanine (F), and alanine (A) were significantly more abundant in the Ksucc peptides. Non-Ksucc amino acids were abundant in arginine (R), leucine (L), and glutamate (E). Meanwhile, lysine (K) was enriched in different positions of Ksucc and non-Ksucc peptides. Therefore, we believed that the differences between these two peptides could be used as a way to distinguish them.

## 4 Conclusion

In this study, Deep_KsuccSite, a novel and effective predictor for predicting Ksucc sites, was developed. Considering the EGAAC, APAAC, CTDC, and Embedding Encoding of proteins, Deep_KsuccSite constructed two base classifiers based on CTDC, the combination of EAGGC and APAAC using 1D-CNN, and a base classifier based on embedding encoding using 2D-CNN, and then voted on those three base classifiers. K-fold cross-validation and independent tests showed that Deep_KsuccSite could be used as a powerful tool to assist in identifying Ksucc sites. In addition, the ablation experiment results based on voting, feature combination, and model architecture showed that Deep_KsuccSite could leverage information from different features to build an effective classifier. The code involved in this study was freely available at https://github.com/flyinsky6/Deep_KsuccSite. In the future, we will carry out further research in three aspects. First of all, the introduction of more protein feature representations to the PTM prediction field, such as protein structure information, evolution information, more physical and chemical properties, etc., will be conducted. For some protein structures that have not been identified yet, we can use the prediction results of SPIDER3 (Heffernan et al., 2017), PSRSM (Zhao et al., 2020), or Nnessy (Krieger and Kececioglu, 2020). Secondly, advanced techniques from natural language processing (NLP) can be introduced to extract protein features, such as Transformer and Bert (Vaswani;, 2017). Many feature embedding methods from the NLP domain have been proved to have good applications in the bioinformatics domain, especially in feature extraction (Ofer et al., 2021). Finally, more effective and interpretable models will be explored in both traditional machine learning and deep learning fields in order to facilitate the understanding of the biological meanings of the prediction results.

## Data Availability

The original contributions presented in the study are included in the article/Supplementary Material, further inquiries can be directed to the corresponding authors.
